# Metallothionein (MT) -I and MT-II Expression Are Induced and Cause Zinc Sequestration in the Liver after Brain Injury

**DOI:** 10.1371/journal.pone.0031185

**Published:** 2012-02-17

**Authors:** Michael W. Pankhurst, David A. Gell, Chris W. Butler, Matthew T. K. Kirkcaldie, Adrian K. West, Roger S. Chung

**Affiliations:** 1 Menzies Research Institute Tasmania, University of Tasmania, Hobart, Tasmania, Australia; 2 Department of Anatomy, University of Otago, Dunedin, New Zealand; 3 School of Medicine, University of Tasmania, Hobart, Tasmania, Australia; University of North Dakota, United States of America

## Abstract

Experiments with transgenic over-expressing, and null mutant mice have determined that metallothionein-I and -II (MT-I/II) are protective after brain injury. MT-I/II is primarily a zinc-binding protein and it is not known how it provides neuroprotection to the injured brain or where MT-I/II acts to have its effects. MT-I/II is often expressed in the liver under stressful conditions but to date, measurement of MT-I/II expression after brain injury has focused primarily on the injured brain itself. In the present study we measured MT-I/II expression in the liver of mice after cryolesion brain injury by quantitative reverse-transcriptase PCR (RT-PCR) and enzyme-linked immunosorbent assay (ELISA) with the UC1MT antibody. Displacement curves constructed using MT-I/II knockout (MT-I/II^−/−^) mouse tissues were used to validate the ELISA. Hepatic MT-I and MT-II mRNA levels were significantly increased within 24 hours of brain injury but hepatic MT-I/II protein levels were not significantly increased until 3 days post injury (DPI) and were maximal at the end of the experimental period, 7 DPI. Hepatic zinc content was measured by atomic absorption spectroscopy and was found to decrease at 1 and 3 DPI but returned to normal by 7DPI. Zinc in the livers of MT-I/II^−/−^ mice did not show a return to normal at 7 DPI which suggests that after brain injury, MT-I/II is responsible for sequestering elevated levels of zinc to the liver. Conclusion: MT-I/II is up-regulated in the liver after brain injury and modulates the amount of zinc that is sequestered to the liver.

## Introduction

Metallothionein (MT) is a 6–7 kDa, cysteine rich, metal binding protein that has been shown to be neuroprotective during central nervous system (CNS) insults in studies utilising transgenic MT-I over-expressing animals [1]–[3] and MT-I/II^−/−^ mice [4]–[11]. Interestingly, it is not MT-III, the brain-specific isoform of MT, that provides neuroprotection [12] but the MT-I and MT-II isoforms that provide the most neuroprotection after brain injury. The MT-I and MT-II isoforms are often considered as a single species (MT-I/II) due to their high homology and the inability of primary antibodies to differentiate between the two forms. The mechanism by which MT-I/II imparts protection to the injured CNS is yet to be fully elucidated.

MT-I/II is expressed in many organs throughout the murine body [13]. Numerous studies have shown that after brain injury, the level of MT-I/II expression in the brain is increased [5], [6], [14]–[17]. MT is chiefly a cytoplasmic protein but increased levels have been observed in the blood of brain injured patients [18]. The expression levels of MT-I/II in other organs after brain injury have not been reported previously and the origin of the MT found in the blood has not been determined. Up-regulation of MT-I/II expression in the liver occurs in response to many stressful stimuli such as burn injury [19]–[21], restraint stress [22], [23], zinc challenge [24], [25], fasting and lipopolysaccharide challenge [26], [27]. The induction of liver MT-I/II expression has been shown to cause increases in hepatic zinc content, a response that does not occur in MT-I/II^−/−^ mice [19]–[21], [24], [25]. Therefore, it appears that the induction of hepatic MT-I/II expression results in the sequestration of zinc to the liver. Zinc sequestration from the plasma is a characteristic of the acute phase response which is typically induced by the cytokine interleukin(IL)-6 [28]. MT-I/II expression is induced by increased intracellular zinc concentration, glucocorticoids and IL-6 [29] which indicates that MT-I/II expression may occur in conjunction with the acute phase response.

Altered zinc homeostasis [30] and raised concentrations of IL-6 in serum [31] have been observed in patients suffering the early stages of brain injury. The process of hepatic MT-I/II mediated zinc sequestration has been proposed to explain these alterations in plasma zinc concentrations [32] but hepatic MT-I/II expression has not been experimentally quantified after brain injury. There is some evidence that systemic zinc status may affect the outcome of brain injury because rats with dietary zinc deficiency preceding experimental brain injury have greater microglial activation and neuron death compared to injured rats on zinc-sufficient diets [7], [33]. There is also a positive association between zinc supplementation after hospital admission and neurologic recovery rate in head injured patients [34].

The aim of this study was to determine whether brain injury in mice causes an increase in hepatic MT-I/II expression and whether any increase in hepatic MT-I/II results in sequestration of zinc to the liver. MT-I/II expression was measured by quantitative reverse-transcriptase PCR (RT-PCR) and enzyme-linked immunosorbent assay (ELISA). The study utilised a MT-I/II^−/−^ mouse strain that still produces MT-I and MT-II mRNAs but premature stop codons in the open-reading-frame result in production of greatly truncated peptides consisting of 10 and 15 amino acids from the N-terminus, respectively [35]. This allowed for liver zinc content after brain injury to be measured in a mouse without fully functional MT-I/II protein.

## Materials and Methods

### Animals

All procedures involving animals were approved by the Animal Experimentation Ethics Committee of the University of Tasmania and were consistent with the Australian Code of Practice for the Care and Use of Animals for Scientific Purposes (Permit number: A9836). 129SI/SvImJ (wild type) mice and 129S7/SvEvBrd-*Mt1^tm1Bri^ Mt2^tm1Bri^*/J (MT-I/II^−/−^) mice [35] were obtained from Jackson Laboratories. Male mice were housed with food and water *ad libitum* with 12/12 hour light/dark cycling. Mice were divided evenly into groups for the time points of 0, 1, 3 and 7 days post-injury (DPI) and were housed in individual cages for at least 7 days prior to surgery.

### Cryolesion brain injury and sham surgery

The cryolesion injury was conducted according to the method of Ling *et al.* [36]. Briefly, animals were anaesthetised for surgery with 3% isoflurane in oxygen, delivered via muzzle mask. A 2–3 mm incision was made in the skin above the midline of the skull. A steel rod, 3 mm in diameter and 50 mm long that had been cooled to equilibrium in liquid nitrogen, was held against the skull for 6 seconds, 4 mm anterior from lambda and 2 mm right of the midline. The incision was then sutured and the animal was allowed to recover back in its original cage. Less than 1% of animals showed signs of seizure within the first 24 hours after the application of the cryolesion injury. These animals were euthanized and excluded from the study. Zero time-point animals were housed identically to injured mice but did not undergo surgery before euthanasia and tissue collection. Sham surgery was conducted in an identical manner to cryolesion surgery except that the steel rod was not cooled in liquid nitrogen before the procedure.

### Quantitative reverse-transcriptase PCR (RT-PCR)

Anaesthetised mice were transcardially perfused with PBS. The cryolesion injury site was dissected out of the brain using a 3 mm biopsy punch and a liver sample was obtained by dissection and both samples were frozen in liquid nitrogen. Liver samples were first ground to a fine powder under liquid nitrogen, brain samples were homogenised whole. Brain and liver samples were homogenised by Ultra-Turrax mechanical homogenizer (IKA) in TRI reagent (Sigma) and RNA was extracted according to manufacturer's protocol. Reverse transcription with the Superscript-III reverse transcriptase system (Invitrogen) and quantitative PCR with Quantitect SYBR green (Qiagen) was conducted according to the method of Brettingham-Moore *et al.* [37]. Oligonucleotide primers are detailed in table 1. The MT-I and MT-II primer sets were designed to bind to the cDNA for the transcripts from both wild type and MT-I/II^−/−^ mice, which still produce MT-I and MT-II transcripts but have premature stop codons inserted to prevent complete protein translation. Standard curves were created using known quantities of each PCR product and were used to determine the original cDNA copy number at an arbitrary fluorescence threshold (C_T_). β-actin mRNA was used as the house keeping gene and MT-I and MT-II mRNA copy numbers was divided by β-actin copy number, to standardise the data set.

**Table 1:** Oligonucleotide primer sets used for quantitative RT-PCR with genbank accession numbers.

Primer		Sequence (5′ - 3′)	Accession No.
β-actin	Fwd	GTCCACCTTCCAGCAGATGT	NM_007393.3
	Rev	AGGGAGACCAAAGCCTTCAT	
MT-I	Fwd	GCTGTCCTCTAAGCGTCACC	NM_013602.3
	Rev	AGGAGCAGCAGCTCTTCTTG	
MT-II	Fwd	CAAACCGATCTCTCGTCGAT	NM_008630.2
	Rev	AGGAGCAGCAGCTTTTCTTG	

### Protein homogenisation

Brain biopsies and liver samples were ground to powder under liquid nitrogen and homogenised in 150 mM NaCl, 20 mM Tris-HCl, 1% Igepal, pH 7.6 with EDTA-free Halt-protease inhibitor cocktail (Thermo Scientific) with an Ultra-Turrax mechanical homogenizer (IKA). Samples were centrifuged at 10 000*g* for 10 minutes and the supernatant was retained for assay. Protein concentration was obtained by Bradford assay [38].

### UC1MT competitive ELISA

MT-IIA (HPLC-purified rabbit MT-IIA saturated with 7 Zn^2+^ ions per molecule, Bestenbalt, Estonia) was coated to a Nunclon delta surface 96-well microplate (Nunc) in 50 mM Na_2_CO_3_ solution at 4°C overnight on an orbital shaker. All subsequent stages took place at room temperature. Following a 5 minute rinse in wash buffer consisting of 0.05% Tween-20 (Sigma) in PBS, wells were blocked with 150 µl casein solution (2.5%, pH 7.4) for 30 minutes. The wells were washed again in wash buffer for 5 minutes. Mouse tissue homogenates or plasma samples were diluted in wash buffer to achieve a protein concentration of 0.1 mg/ml and standard MT-IIA solutions were made up in wash buffer. Samples and standards were applied to the plate in triplicate or quadruplicate in 50 µl aliquots. Primary antibody (UC1MT mouse anti-MT-I/II, Assay designs) was diluted 1∶5000 (40 ng/ml, final concentration) in ELISA wash buffer and applied to sample- or standard-containing wells for 1 hour. The plate was rinsed 3 times with wash buffer. Secondary antibody (Dako, Goat anti-mouse IgG-horse radish peroxidase (HRP) conjugate) was diluted 1∶2000 in ELISA wash buffer and applied to each well in 50 µl aliquots for 1 hour. The plate was rinsed 3 times with wash buffer. 50 µl TMB peroxidase substrate (KPL) was applied for 1 hour. The reaction was terminated with 50 µl of 1 M phosphoric acid and the absorbance was measured at 450 nm. Calculation of sample MT-I/II concentrations from the standard curve was conducted with 4-parameter logistic modelling according to the method of Findlay and Dillard [39]. Displacement curves were generated by serial dilution of MT-IIA standard solutions in tissue homogenate from MT-I/II^−/−^ mice. Several curves were created for each tissue type with varying concentrations of total protein.

### Direct UC1MT ELISA to compare MT-IIA and MT-III cross-reactivity

Microplate wells were coated with 50 µl of standard curve solutions for MT-IIA and MT-III (HPLC-purified human MT-III saturated with 7 Zn^2+^ ions per molecule, Bestenbalt, Estonia) which were made up in 50 mM Na_2_CO_3_ and incubated overnight at 4°C on an orbital shaker. Following a 5 minute rinse in wash buffer, wells were blocked with 150 µl casein solution (2.5%, pH 7.4) for 30 minutes. The wells were washed again in wash buffer for 5 minutes. UC1MT primary antibody was diluted 1∶1000 in ELISA wash buffer and applied to wells and incubated for 1 hour. The plate was rinsed for 3 times with wash buffer. HRP-conjugated secondary antibody was diluted 1∶2000 in ELISA wash buffer and applied to each well in 50 µl aliquots for 1 hour. After rinsing with wash buffer, TMB peroxidase substrate (KPL) was incubated in the wells in 50 µl aliquots for 1 hour. The reaction was terminated by addition of 50 µl of 1 M phosphoric acid and the absorbance was measured at 450 nm.

### Radioimmunoassay for corticosterone

Blood was obtained from mice at 0, 1, 3 or 7 DPI. Mice were anaesthetised by being placed in a chamber containing 5% isoflurane in oxygen. As soon as anaesthesia was achieved, 0.5 ml of blood was obtained via cardiac puncture with syringes containing 20 µl of 5000 units/ml heparin solution (Sigma). Plasma was obtained by centrifugation at 14 000×*g* for 5 minutes. Assay of plasma corticosterone was conducted as described in Pankhurst *et al.* [40]. Standard solutions of unlabelled corticosterone were prepared by serial dilution in assay buffer and were added directly to assay tubes. A standard curve was generated to calculate the concentration of corticosterone in each sample. All samples and standards were assayed in duplicate. Extraction efficiency for mouse plasma was determined by adding a known quantity of ^3^H-corticosterone to plasma samples pooled from several mice and the assay concentrations were adjusted accordingly.

### Liver zinc assay by atomic absorption spectroscopy

Liver samples from uninjured and injured mice were dissected out and freeze-clamped in liquid nitrogen. Each sample was ground to a fine power under liquid nitrogen with a mortar and pestle. The powdered liver was homogenised in 1 ml of MilliQ (Millipore) water with a Potter-Elvehjem homogeniser. The homogenate was transferred to a pre-weighed tube and was lyophilised. The gross weight of the tube was measured after lyophilisation to calculate the net weight of the sample. Lyophilised liver samples were dissolved in 3.5 ml of 70% nitric acid (Trace select, Fluka) by heating to 70°C for 1 hour. The samples were diluted with 3 ml of MilliQ water and 0.5 ml of 30% hydrogen peroxide to fully oxidise thiols (Trace select, Fluka). Centrifugation at 2000×*g* for 20 minutes was required to remove a small quantity of insoluble matter. Zinc concentration in each sample was assayed on an atomic absorption spectrometer (GBC Avanta Σ). Zinc concentration of the sample solutions was determined by comparison to the absorbance of zinc sulphate standard solutions prepared in 35% nitric acid and 2.14% hydrogen peroxide in MilliQ water.

### Statistical analysis

Homogeneity of variances between groups within each data set was determined with Levene's test. The Box-Cox test was used to determine the appropriate transformation for data sets with heterogeneous variances between groups. Comparisons of MT-I and MT-II mRNA, MT-I/II protein expression and liver zinc content were conducted by 1-way ANOVA with Tukey's B post-hoc test. The comparison of corticosterone between wild type and MT-I/II^−/−^ mice was conducted with 2-way ANOVA with Tukey's B post-hoc test on the factors of time after injury and strain of mouse. For all experiments, differences were considered statistically significant where *p*<0.05.

## Results

### UC1MT ELISA optimisation and validation

**Figure 1:**
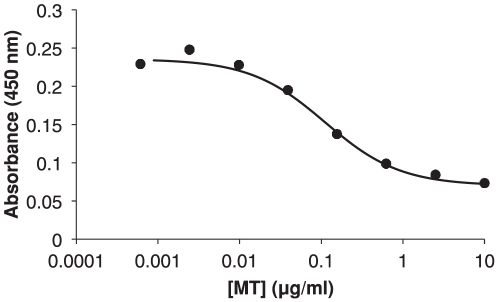
Standard curve generated by the UC1MT competitive ELISA with 4-parameter logistic curve fitted. Each point shown is the average of 4 quadruplicate standards.

We optimised the MT-I/II ELISA procedure published by Emeny *et al.* [41]. By trialling different coating concentrations of recombinant MT-IIA and different antibody dilutions, it was determined that the optimal coating concentration for the microplate was 100 ng/ml MT-IIA. Optimal primary antibody dilution of 1∶5000 (40 ng/ml IgG final concentration) and secondary antibody dilution of 1∶2000 (0.5 µg/ml final IgG concentration) yielded the highest sensitivity in the ELISA (data not shown). Figure 1 shows the standard curve of the ELISA with 4-parameter logistic curve fitted. A cut-off for the detection limit of was set at 10 ng/ml MT and yet this required 500-fold lower levels of primary antibody than the procedure of Emeny *et al.* [41]. The coefficient of variation was used to determine the range of the intra-assay variability of the ELISA and was calculated to be 3.46–10.33%cv.

**Figure 2:**
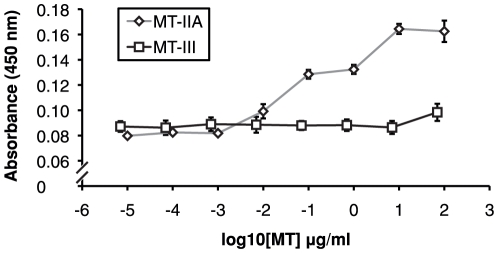
Cross-reactivity of the UC1MT antibody for MT-III was tested by direct ELISA. Comparison of the standard curves for MT-IIA (blue lines) and MT-III (red lines) demonstrate UC1MT has very little if any cross-reactivity for MT-III. Data are expressed as the mean of triplicate measurements (error bars = SEM).

When the UC1MT antibody was initially characterised for its ability to detect MT-I and MT-II by ELISA [42], the discovery of MT-III was relatively recent and no test for cross-reactivity of MT-III with this antibody has since been published. It was necessary to demonstrate that the UC1MT ELISA is specific for MT-I/II and does not cross-react with MT-III, the brain-specific MT isoform (figure 2). It is apparent from the curves that the UC1MT antibody displays little cross reactivity with MT-III.

**Figure 3:**
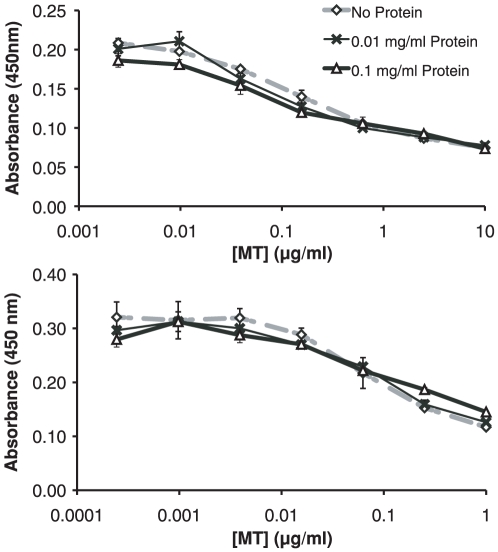
Displacement curves for MT-IIA in MT-I/II^−/−^ mouse brain homogenate (A) and MT-I/II^−/−^ mouse liver homogenate (B). Displacement curves constructed in solutions with protein content of 0.01 mg/ml and 0.1 mg/ml are parallel to the standard curve constructed in PBS. Therefore, no matrix effects were observed at these concentrations, in these tissues (n = 3, error bars = SEM).

Displacement curves were set up to determine if matrix effects that could interfere with the ELISA were present in mouse brain or liver tissue homogenates (figure 3). Such curves have not been published previously for the UC1MT ELISA technique. The term “matrix effects” relates to substances in complex biological samples that do not directly cause false-positive detection in an immunoassay but have the capacity to displace the antibody-antigen interaction [43], [44]. Displacement curves are similar to standard curves except that serial dilution of the analyte is performed in analyte-free matrix, in this case tissue homogenate from MT-I/II^−/−^ mouse brain and liver was used. Using MT-I/II-free brain and liver homogenates with total protein concentration of 0.1 mg/ml or 0.01 mg/ml, we observed no deviation of the slope of the displacement curves from the standard curve which indicates that no matrix effects are present under these assay conditions, in these tissues. We have determined that concentrations higher than 0.1 mg/ml begin to interfere with the assay in most of the tissue types tested.

### MT-I and MT-II induction in liver post-brain injury

**Figure 4:**
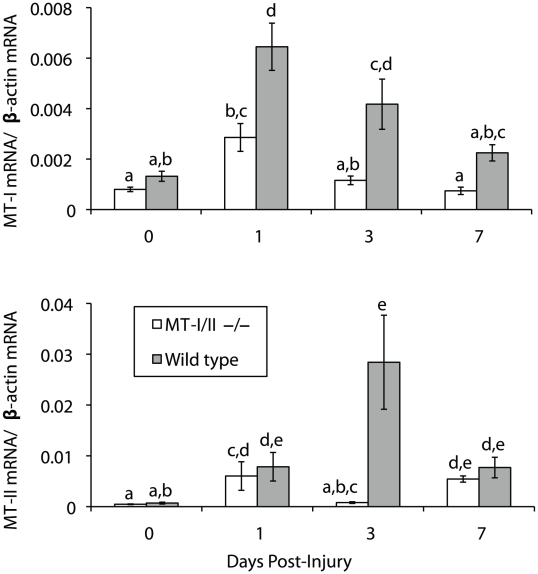
Expression of MT-I and MT-II mRNA in the liver of wild type and MT-I/II^−/−^ mice after brain injury was quantified by RT-PCR. (**A**) MT-I mRNA expression showed its greatest increase at 1 DPI and 3 DPI in wild type mice. (**B**) MT-II mRNA was increased at 1 DPI in wild type mice but was at peak levels at 3 DPI. MT-I/II^−/−^ mice were unable to increase MT-I and MT-II mRNA levels to the same extent as wild type mice. Groups that share lower case letters are not significantly different from each other (for both graphs; n = 6–7, error bars = SEM).

**Figure 5:**
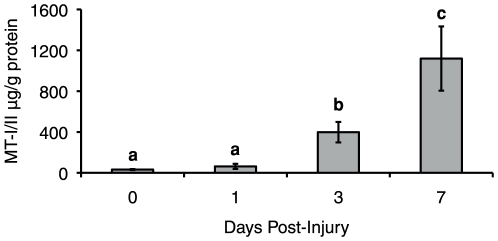
Liver MT-I/II protein levels after cryolesion injury to the brain were assayed by UC1MT ELISA in wild type mice. Hepatic MT-I/II protein levels were not increased until 3 DPI and showed a further increase at 7 DPI. Groups that share lower case letters are not significantly different from each other (n = 7, error bars = SEM). signalling mechanism is involved.

In the site of the brain injury, expression of MT-I and MT-II mRNA and MT-I/II protein was increased within 1 DPI (data not shown), as has been observed previously [5], [6], [14]–[17]. Quantitative RT-PCR revealed that liver MT-I and MT-II mRNA increases after brain injury (figure 4). Wild type mice showed a 4.9 fold increase in MT-I mRNA at 1 DPI in the liver, followed by subsequent decreases at 3 and 7 DPI. MT-II mRNA was significantly increased at 1 DPI but was maximal at 3 DPI with a 40.4 fold increase in expression over uninjured levels. Interestingly, increases in MT-I/II protein levels, as determined by competitive ELISA, were delayed and did not show significant increases until 3 DPI with maximal protein expression at 7 DPI (figure 5). The possibility of MT-I/II protein increases from zero to 1 DPI could not be investigated because the quantities detected at both of these time points were less than or equal to the detection limit of the assay.

The nature of the targeted disruption of the MT-I and MT-II genes in the MT-I/II^−/−^ mouse strain allows for the measurement of gene transcription in the absence of expression of the full-length proteins. In MT-I/II^−/−^ mice the level of MT-I and MT-II mRNA in the liver after brain injury is reduced compared to wild type mice (figure 4).

### Plasma corticosterone concentration increases after cryolesion brain injury

**Figure 6:**
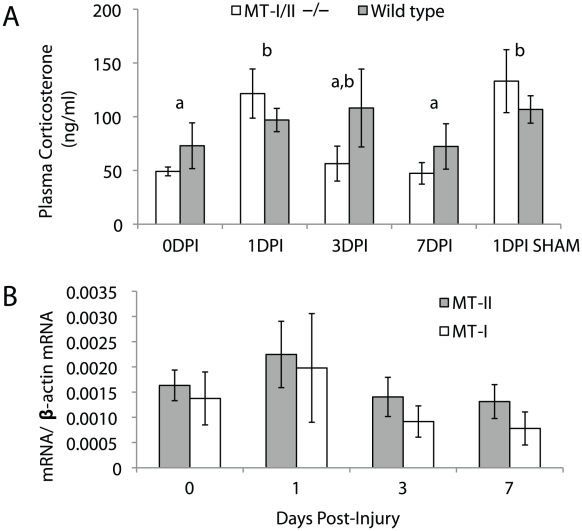
Corticosterone concentrations in plasma after cryolesion injury to the brain were assayed by RIA in wild type and MT-I/II^−/−^ mice (A). No significant differences were found between the mouse strains. There was a significant increase in plasma corticosterone after cryolesion injury and sham surgery to a similar extent. Sham surgery does not induce a significant change in hepatic MT-I or MT-II mRNA expression (**B**). Time points that share letters are not significantly different (n = 5, error bars = SEM).

The glucocorticoid, corticosterone, is the primary stress hormone produced by the adrenal gland in rodents, and has an analogous role to cortisol in humans. Thus, we hypothesised that corticosterone may influence hepatic MT-I/II synthesis as part of a systemic response to brain injury. To test this in our model, plasma corticosterone levels were assayed by radioimmunoassay in both wild type and MT-I/II^−/−^ mice, with injury, without injury and with sham injury surgery (figure 6A). No significant differences in plasma corticosterone were found between wild type and MT-I/II^−/−^ mice before or after injury as determined by 2-way ANOVA. However, it was determined that there were significant and comparable increases in plasma corticosterone concentrations in cryolesioned and sham-injured animals. This indicates that animal handling is the most likely responsible for the increases in plasma corticosterone rather than the brain injury. Quantitative RT-PCR was carried out on liver samples from sham-injured animals but no significant increases in MT-I or MT-II mRNA expression were observed after sham surgery, despite the ability of the procedure to increase plasma corticosterone (figure 6B). In summary, the increases in plasma corticosterone after brain injury or sham surgery are not sufficient to induce hepatic MT-I/II expression alone and it is likely that another process is responsible for the increased hepatic expression of MT-I/II after brain injury.

### Liver zinc post-injury

**Figure 7:**
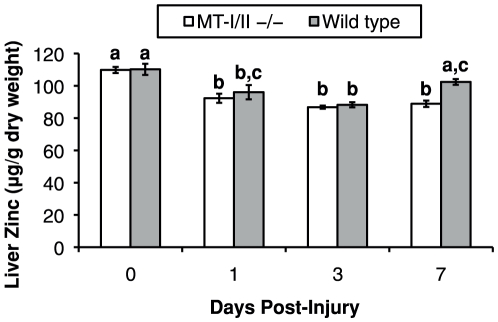
Liver zinc content was measured by atomic absorption spectroscopy. Liver Zinc content was decreased in both wild type and MT-I/II^−/−^ mice at 1 and 3 DPI. 1-way ANOVA revealed significantly higher hepatic zinc in wild type than MT-I/II^−/−^ mice at 7 DPI. Groups that share lower case letters are not significantly different from each other (n = 5–6, error bars = SEM).

Liver zinc was assayed by atomic absorption spectroscopy in MT-I/II^−/−^ and wild type mice to determine if hepatic MT-I/II sequesters zinc after brain injury. In the absence of injury there is no significant difference in the liver zinc content between the two mouse strains, indicating that MT-I/II normally makes a minor contribution to total zinc binding. Brain injury caused a slight decrease in liver zinc content at 1 and 3 DPI in both strains of mouse (figure 7), suggesting release of zinc from non-MT-I/II source. However, at 7 DPI significant differences between wild type and MT-I/II^−/−^ mice were observed. In the wild-type mice we saw a recovery in zinc content to a level that was not significantly different to the level seen pre-injury. This increase in liver zinc content at 7 DPI corresponds precisely to the period when dramatic increases in MT-I/II protein levels were observed (figure 5). In contrast, no significant recovery in hepatic zinc was observed for MT-I/II^−/−^ mice at 7 DPI which strongly implies that MT-I/II is responsible for this process.

## Discussion

The observations presented herein are consistent with a model whereby zinc is released from cellular stores in the liver following brain injury, and that increased MT-I/II expression then functions to return zinc to the liver. In human studies it has been found that serum zinc levels are decreased upon admission of head injury patients to hospital [30]. Zinc excretion via urine was found not to be the cause and it was later hypothesised that MT-I/II expression in the liver was sequestering zinc from the plasma [32]. Our data suggest that, in mice, zinc is initially released from the liver and that MT-I/II expression is then up-regulated in order to restore hepatic zinc to pre-injury levels. Because the liver is a relatively large organ, this response may also have the ability to affect zinc availability in other organs. It has been shown previously that MT-I/II induction by the liver has the ability to buffer sudden, systemic increases in free zinc after zinc injection [24]. In the present study, the zinc composition of the diet remained constant but it is clear that zinc is becoming mobilised from the liver. The effect that disruption to zinc homeostasis has on the progression of brain injury is not well understood.

The factors that directly induce MT-I and MT-II mRNA expression have been identified as increased intracellular free zinc concentration via interaction with metal transcription factor-1 (MTF-1), IL-6 signalling and glucocorticoid receptor activation [29]. The data in the present study indicated that the glucocorticoid, corticosterone, was unlikely to be responsible for inducing an increase in MT-I or MT-II mRNA expression in the liver. *In vitro*, glucocorticoids are capable of increasing levels of MT-I/II mRNA in hepatocyte cultures [45]. However, *in vivo* experiments investigating the induction of hepatic MT-I/II by restraint stress suggest that glucocorticoids are responsible for altering the translation of mRNA into protein but that IL-6 signalling is the factor responsible for inducing MT-I/II mRNA, not glucocorticoids [22]. It remains a possibility that corticosterone enhances the expression of hepatic MT-I/II synthesis after brain injury, even if it is not solely responsible for MT induction. The cytokine IL-6 has been found to be increased in the serum of head-injured patients [31]. Using this cryolesion injury model, increased IL-6 mRNA expression can be detected at the site of brain injury but IL-6 protein was not readily detected in plasma by cytokine assay, nor was there *de novo* synthesis of IL-6 mRNA detected in the liver (unpublished observations). Therefore IL-6 is unlikely to play a role in the induction of hepatic MT-I/II in our brain injury model.

By process of elimination, zinc remains as the most likely signal responsible for inducing hepatic MT-I/II expression after brain injury, further supporting the hypothesis that brain injury disrupts systemic zinc homeostasis. We provide the following hypothesis to explain the increases in MT-I and MT-II mRNA expression despite decreases in total zinc. Zinc is transported out of cells as free ions but the majority of zinc in cells is bound to zinc-binding proteins. Oxidative stress can displace zinc from metalloproteins [46], effectively increasing the free zinc concentration inside the affected cell. Free zinc would be susceptible to efflux transport, but would also be available to bind to metal-transcription factor-1, a complex which facilitates MT-I and MT-II gene transcription [reviewed in 29]. Head injury to rats has been shown to induce a whole body oxidative stress within 15 minutes of the injury [47]. MT-I/II itself, is a zinc-binding metalloprotein and it is susceptible to oxidation leading to zinc liberation [48], [49]. Liberation of zinc from proteins and subsequent transport from hepatocytes is consistent with the decreased hepatic zinc content observed at 1 DPI in the present study. Hence, an oxidative mechanism provides one hypothesis to explain brain injury mediated decreases in total hepatic zinc levels with simultaneous increases in MT-I and MT-II mRNA expression.

High expression of MT-I/II is thought to decrease cytoplasmic concentrations of free zinc. However, it has been shown that under normal physiological conditions, the amount of zinc bound to MT-I/II is below maximum capacity [50]. For hepatic MT-I/II to be involved in zinc sequestration, the ratio of MT-I/II to cellular zinc is expected to be much higher to create a sufficient diffusion gradient to favour the entry, and retention, of zinc in the hepatic cytoplasm. High levels of MT-I/II protein could also provide negative feedback for MT-I and MT-II mRNA expression by binding free-zinc and limiting the availability of cytoplasmic zinc to MTF-1. This would explain the low levels of mRNA transcript observed at 7 DPI, despite high levels of MT-I/II protein. The fact that MT-I/II^−/−^ mice have altered expression of MT-I and MT-II mRNA is in accordance with our hypothesis that MT-I/II is one of the storage proteins for zinc that can play a role in its own regulation when zinc is liberated from proteins. However, it warrants mention that cellular processes known collectively as nonsense-mediated mRNA decay exist to remove abnormal transcripts [51] and may be removing the MT-I and MT-II transcripts in MT-I/II^−/−^ mice at a faster rate than normal because of the premature stop codons they contain. Reporter assays in cells isolated from MT-I/II^−/−^ mice may be required in future experiments to determine which of these possibilities is occurring.

In conclusion, we have shown that brain injury, like many severe perturbations to an animal, causes an increase in hepatic MT-I/II expression which leads to sequestration of zinc to the liver. This shuttling of zinc may affect the hepatic processes after brain injury but may also have implications for systemic zinc availability after brain injury. Given that the mechanism by which MT-I/II is neuroprotective after brain injury is unclear, these findings introduce the possibility that MT-I/II expressed outside the central nervous system could impact upon brain injury.
